# Supplementary material for: A set of multi-entry identification keys to African frugivorous flies (Diptera, Tephritidae)
Source: Zookeys. 2014 Jul 24;(428):97–108. doi: 10.3897/zookeys.428.7366 (PMC4143993; doi:10.3897/zookeys.428.7366)
Supplement: Supplementary material 6 — Key to Ceratitis [file zookeys-428-097-s006.zip › SF6_ZooKeys_key to Ceratitis/key/SF6_ZooKeys_key to Ceratitis/Media/Html/Ceratitis chirinda.htm]

***Ceratitis
(Pterandrus) chirinda*** **(Hancock)**

*Trirhithromyia
chirinda* Hancock,
1985: 296.

Body
length: 4.3mm; wing length: 4.5mm.

Male.

Head. Antenna dark orange. Arista with long rays (but figure
in Hancock, 1985 shows short rays). Frons convex; yellow, becoming red-brown
anteriorly. Frontal setae well developed. Face yellow, gena darker. Genal seta
and setulae black, latter well developed.

Thorax. Postpronotal lobe whitish,
with black spot. Scutal pattern shiny brownish black, with dark pilosity;
center largely gray-dusted; yellow prescutellar spots present but
inconspicuous. Scapular setae dark. Scutellum yellow, basally with two merged
dark spots, forming a dark border; apically with three merged black spots
extending to basal 0.33 to 0.25, weakly incised. Anepisternum on ventral half
brownish black.

Legs.
Yellowish orange except femora brown, tibiae with basal half partly brown;
without any feathering.

Wing. Bands
brown. Marginal band forming continuous band with anterior part of discal band;
cubital band joining junction of marginal and distal band; medial band absent;
crossvein R-M just proximal to middle of discal cell. Apex of vein R_1_
distal to level of crossvein R-M. Crossvein DM-Cu oblique posterobasally.

Abdomen.
Shiny black. Tergites 2 and 4 across posterior half with silvery band.

Female

Unknown.

(Redescription
after De Meyer & Freidberg, 2006)
